# Multiple peritoneal dissemination of T2 colorectal cancer without lymph node metastases: a case report

**DOI:** 10.1093/jscr/rjaa118

**Published:** 2020-07-31

**Authors:** Yosuke Namba, Yuzo Hirata, Shoichiro Mukai, Toshihiro Nishida, Syo Ishikawa, Azusa Kai, Akihiro Kohata, Syo Okimoto, Seiji Fujisaki, Saburo Fukuda, Mamoru Takahashi, Toshikatsu Fukuda

**Affiliations:** 1 Department of Surgery, Chugoku Rosai Hospital, Kure, Japan; 2 Department of Gastroenterological and Transplant Surgery Applied Life Sciences, Institute of Biomedical and Health Sciences, Hiroshima University, Hiroshima, Japan; 3 Department of Pathology Clinical Laboratory, Chugoku Rosai Hospital, Kure, Japan

## Abstract

Most cases of peritoneal dissemination of colorectal cancers are from T3 or T4 tumors. A 61-year-old woman was admitted for examination of a positive fecal occult blood test. Colonoscopy showed an ascending colon tumor that was diagnosed as an adenocarcinoma with massive submucosal invasion. Imaging modality revealed numerous nodules throughout the abdominal cavity. Peritoneal dissemination of the ascending colon or ovarian cancer and pseudomyxoma peritonei were considered in the preoperative differential diagnoses, and laparoscopic ileocecal resection was performed. Intraperitoneal observation revealed numerous white nodules in the peritoneum, omentum and Douglas fossa. Both the nodules and tumor were diagnosed as mucinous carcinoma based on a pathology report. The tumor invasion depth was limited to muscularis propria, and no regional lymph node metastasis was detected. Peritoneal dissemination of the ascending colon cancer was considered. We report a rare case of multiple peritoneal dissemination of T2 colorectal cancer without lymph node metastases.

## INTRODUCTION

Colorectal cancer (CRC) is a leading cause of cancer-related deaths worldwide, and its incidence has been increasing [1]. The 5-year survival rate of patients with Stage IV CRC is 12.5–17.8% [2, 3]. In particular, peritoneal dissemination cases have poor prognosis. The median cancer-specific survival of single-organ metastases in the peritoneum group was shorter than that of single-organ metastases at a site other than the peritoneum group, and it was equivalent to that of the multiple-organ metastases group [4]. Peritoneal dissemination is present in 4–10% of all CRCs, most of which are T3 or T4 CRCs [5]. Sato et al. reported that the tumor invasion depth was 9.0% for T3 and 91.0% for T4 in patients with peritoneal dissemination of CRC (TNM Classification of Malignant Tumors, seventh edition), and the incidence of lymph node metastases in these patients was 91.0% [6].

**Figure 1:**
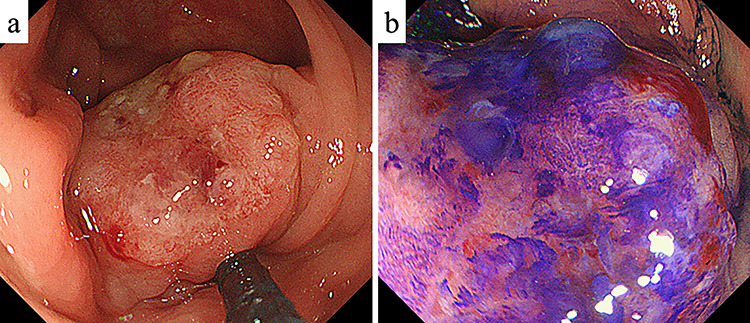
Colonoscopy. (**a**) Colonoscopy revealed a Type I tumor with depression and fullness in the ascending colon; (**b**) according to the Kudo pit pattern classification, this tumor was V_I_ high grade.

To our investigation, no case of multiple peritoneal dissemination of T1 or T2 CRC has been reported. Here, we report a rare case of multiple peritoneal dissemination of T2 CRC without lymph node metastases.

## CASE REPORT

**Figure 2:**
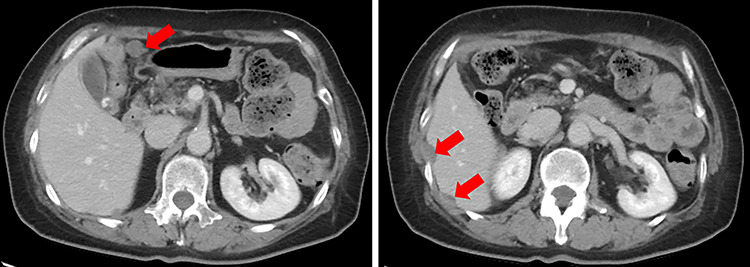
Enhanced CT. Numerous nodules were confirmed throughout the abdominal cavity (red arrows).

**Figure 3:**
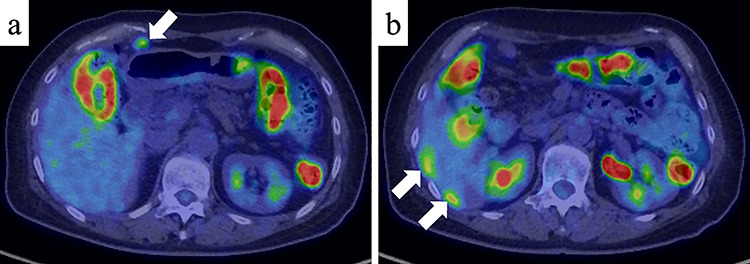
Positron emission tomography and CT (PET/CT) (**a**) PET/CT showed a nodule with mild FDG uptake in the peritoneum (SUVmax = 3.0) (white arrow). (**b**) Two nodules with mild FDG uptake (SUVmax = 3.8 and 4.1, respectively) were confirmed around the liver (white arrows).

A 61-year-old woman was admitted to our hospital for examination of positive fecal occult blood test. She had a medical history of diabetes but no surgical history. Laboratory data showed that complete blood cell count and hepatic and renal functions were normal. The serum carcinoembryonic antigen level was slightly elevated to 8.7 ng/ml (reference value < 5.0 ng/ml), and the carbohydrate antigen 19-9 level was within the normal range. Colonoscopy demonstrated a Type I tumor with depression and fullness in the ascending colon, and it was V_I_ high grade according to the Kudo pit pattern classification (Fig. 1); tissue biopsy was obtained during colonoscopy. This tumor was diagnosed as adenocarcinoma with massive submucosal invasion. Enhanced computed tomography (CT) revealed numerous nodules throughout the abdominal cavity and a small amount of ascites in the pelvic floor (Fig. 2). Ascending colon cancer could not be pointed out. Positron emission tomography and CT revealed a nodule with mild fluorine-18-fluorodeoxyglucose (FDG) uptake in the peritoneum (maximum standardized uptake value (SUVmax) = 3.0) and two nodules with mild FDG uptake (SUVmax = 3.8 and 4.1) around the liver (Fig. 3). Abnormal FDG uptake that showed the primary tumor was not found. Peritoneal dissemination of the ascending colon cancer was considered among the preoperative differential diagnoses. Based on the degree of the FDG uptake, pseudomyxoma peritonei was also differentially diagnosed.

**Figure 4:**
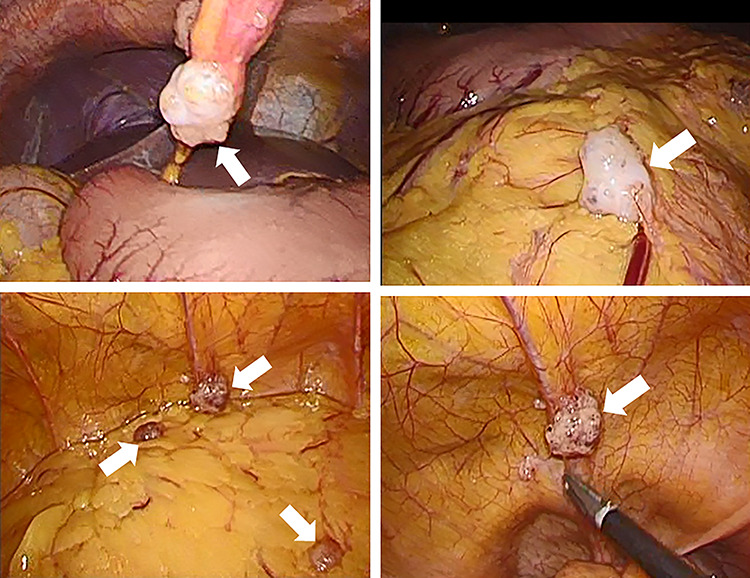
Intraperitoneal observation. Numerous white nodules are found in the peritoneum, omentum and Douglas fossa (white arrows).

**Figure 5:**
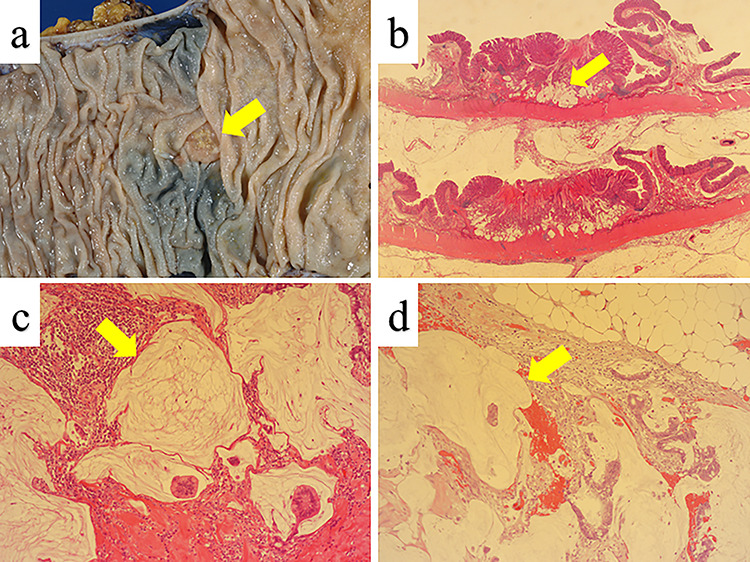
Histopathology. (**a**) The resected specimen showed the depressed mass (1.0 × 1.0 cm) in the ascending colon (yellow arrow). (**b**) The depth of tumor invasion was muscularis propria (yellow arrow) (hematoxylin & eosin [H&E] stain, ×10). (**c** and **d**) The mass in the ascending colon and white nodules are diagnosed as mucinous carcinoma (yellow arrows) (H&E stain, ×40).

Laparoscopic ileocecal resection with D2 lymphadenectomy was performed for both diagnostic and therapeutic purposes. Intraperitoneal observation revealed numerous white nodules in the peritoneum, omentum and Douglas fossa (Fig. 4). Two nodules were biopsied from the peritoneum and omentum, and both nodules were diagnosed as mucinous carcinomas (Fig. 5d). No neoplastic lesions were observed in the ovaries and liver. A depressed mass (1.0 × 1.0 cm) was found in the ascending colon and was diagnosed as mucinous carcinoma, similar to the white nodules based on a pathology report (Fig. 5a–c). Immunostaining also showed that the ascending colon cancer was consistent with the white nodules. The tumor invasion depth was limited to muscularis propria, and no lymph node metastases were detected in the 18 removed regional lymph nodes. No malignant lesions were found in the appendix. Based on these findings, peritoneal dissemination of the ascending colon cancer was considered, and the tumor was classified as fT2N0M1c fStageIVC (TNM Classification of Malignant Tumors, eighth edition).

The postoperative course was uneventful, and FOLFOXIRI plus bevacizumab chemotherapy was performed on postoperative day 29.

## DISCUSSION

In this study, we report about a rare case or multiple peritoneal dissemination of T2 CRC without lymph node metastases.

The common mechanism of peritoneal dissemination is direct dissemination from the primary tumor into the peritoneal cavity, and other routes include lymphatic and hematogenous dissemination [7, 8]. Peritoneal dissemination of T2 CRC, as in this case, is considered to occur because of lymphatic or hematogenous dissemination. However, most routes are direct dissemination into the peritoneal cavity, and peritoneal dissemination of CRC has only been reported from T3 or T4 tumors within our investigation. Sato et al. reported that the tumor invasion depth was 9.0% for T3 and 91.0% for T4 in patients with peritoneal dissemination of CRC [6]. Additionally, another study reported that the tumor invasion depths were 27.7% for T3 and 72.3% for T4 in patients with peritoneal dissemination of CRC [4].

Besides advanced T stage, the risk factors for developing peritoneal metastases from CRC are principally right-sided colon cancer, mucinous adenocarcinoma, patients younger than 70–75 years, emergency surgery at diagnosis, lymph node metastases, synchronous ovarian metastases and incomplete primary tumor resection [9, 10]. In this case, the three factors of right-sided colon cancer, mucinous adenocarcinoma and younger patients are noted. The incidence of lymph node metastases in the peritoneal dissemination of CRC was 91.0% [6], and besides advanced T stage, lymph node metastases may be an important risk factor.

Multiple peritoneal dissemination of T2 CRC without lymph node metastases, as in this case, is very rare. The mechanism of peritoneal dissemination may be lymphatic or hematogenous; however, it is unclear why metastases occurred only in the peritoneum and not in the lymph node or other organs. Thus, further studies are needed.

This case showed that peritoneal dissemination could occur in patients with T2 CRC without lymph node metastases. Furthermore, there are various risk factors besides advantage T stage, and the strategies of examination or treatment should be formulated considering the possibility of multiple peritoneal dissemination of T1 and T2 CRCs.
